# Focused Design of Novel Cyclic Peptides Endowed with GABARAP-Inhibiting Activity

**DOI:** 10.3390/ijms23095070

**Published:** 2022-05-03

**Authors:** Enrico Mario Alessandro Fassi, Mariangela Garofalo, Jacopo Sgrignani, Michele Dei Cas, Matteo Mori, Gabriella Roda, Andrea Cavalli, Giovanni Grazioso

**Affiliations:** 1 Dipartimento di Scienze Farmaceutiche, Università degli Studi di Milano, Via L. Mangiagalli 25, 20133 Milano, Italy; enrico.fassi@unimi.it (E.M.A.F.); matteo.mori@unimi.it (M.M.); gabriella.roda@unimi.it (G.R.); 2 Dipartimento di Scienze del Farmaco, Università di Padova, Via F. Marzolo 5, 35131 Padova, Italy; mariangela.garofalo@unipd.it; 3 Institute for Research in Biomedicine (IRB), Via Chiesa 5, 6500 Bellinzona, Switzerland; jacopo.sgrignani@irb.usi.ch (J.S.); andrea.cavalli@irb.usi.ch (A.C.); 4 Dipartimento di Scienze della Salute, Università degli Studi di Milano, Via A. di Rudinì 8, 20146 Milano, Italy; michele.deicas@unimi.it

**Keywords:** peptide, GABARAP inhibitors, autophagy, cancer, Atg8, LIR motif, PC-3

## Abstract

(1) Background: Disfunctions in autophagy machinery have been identified in various conditions, including neurodegenerative diseases, cancer, and inflammation. Among mammalian autophagy proteins, the Atg8 family member GABARAP has been shown to be greatly involved in the autophagy process of prostate cancer cells, supporting the idea that GABARAP inhibitors could be valuable tools to fight the progression of tumors. (2) Methods: In this paper, starting from the X-ray crystal structure of GABARAP in a complex with an AnkirinB-LIR domain, we identify two new peptides by applying in silico drug design techniques. The two ligands are synthesized, biophysically assayed, and biologically evaluated to ascertain their potential anticancer profile. (3) Results: Two cyclic peptides (WC8 and WC10) displayed promising biological activity, high conformational stability (due to the presence of disulfide bridges), and K_d_ values in the low micromolar range. The anticancer assays, performed on PC-3 cells, proved that both peptides exhibit antiproliferative effects comparable to those of peptide K1, a known GABARAP inhibitor. (4) Conclusions: WC8 and WC10 can be considered new GABARAP inhibitors to be employed as pharmacological tools or even templates for the rational design of new small molecules.

## 1. Introduction

Autophagy plays a fundamental role in cellular, tissue, and organismal homeostasis. Metabolic stress (starvation and hypoxia) or the presence of dangerous cellular components, including dysfunctional organelles, intracellular microbes, and pathogenic proteins, can activate this pathway. Briefly, a multistep process, starting with the assembly of the phagophore, mediates the sequestration of organelles and proteins into the autophagosome. Its subsequent fusion with a lysosome leads to the formation of the autolysosome, in which the autophagosome content is degraded by lysosomal hydrolases [1].

More than 50 proteins (called Atgs) are involved in the autophagy machinery, but those responsible for the formation of the autophagosome and for cellular trafficking are members of the Atg8 family. In mammals, Atg8 proteins (mAtg8) are further divided into two subfamilies: GABARAP (GABA-A receptor-associated protein) and MAP1LC3 (microtubule-associated protein 1 light chain 3), or simply LC3. The former comprises GABARAP, GARAPL1, and GABARAPL2, while the latter includes LC3A (with the two splicing variants LC3Aα and LC3Aβ), LC3B, LC3B2, and LC3C [2]. Proteins belonging to the same subfamily share a high-sequence homology and play similar physiological roles at the intracellular level. The GABARAP subfamily seems fundamental for the closure of the autophagosome and for the recruitment of the autophagy players, while LC3 proteins appear to be mainly involved in the cargo recruitment process.

Disfunctions in the mAtg8 system have been identified in various conditions, including neurodegenerative diseases, cancer, and inflammation; however, the role of each Atg8 component in cancer is still undetermined and controversial. In the early stage of tumorigenesis, a high level of autophagy proteins is considered a good prognosis factor, since GABARAP is downregulated in renal and breast cancers, and hepatocarcinoma [2]. Conversely, high levels of GABARAP have been detected in colorectal and thyroid cancers. Moreover, cancer cells use autophagy to survive to several antitumor drugs. Some reports indicate that the efficacy of radio- and chemotherapy is strongly influenced by the effective modulation of the autophagy process [3,4]. Additionally, it has been demonstrated that the expression level of mAtg8 proteins is strictly related to the tumor development, stage, and type [2].

Furthermore, it has been shown that the autophagy machinery is often inefficient in prostate cancer cells, due to a reduction in catabolic pathways [5]. In 2012, He et al. [6] suggested that the apoptotic effects of some agents, such as the TNF-related apoptosis-inducing ligand (TRAIL), was enhanced by inhibiting pharmacological autophagy [7]. Therefore, these processes seem to play a pivotal role in the regulation of the death/survival balance in prostate cancer cells. In 2016, Engedal et al. proved that GABARAP-subfamily proteins are strongly involved in autophagy mechanisms in prostate cancer [8], supporting the idea that GABARAP inhibitors could be valuable tools to fight the progression of this disease.

To the best of our knowledge, the known GABARAP inhibitors are essentially proteins or peptides of various sizes. Among them, the small synthetic peptide K1 (DATYTWEHLAWP) is one of the most active candidates, showing a K_d_ value close to 390 nM (data measured by Surface Plasmon Resonance experiment, SPR) [9]. Additionally, an interesting natural GABARAP binder is AnkirinB (AnkB), a 440 kDa neural-specific protein expressed in unmyelinated axons. Similar to other proteins involved in the autophagy machinery, AnkB has an LC3 interacting region (LIR), a small area containing four conserved amino acids. These residues can be briefly represented as a sequence of “X_0_–X_1_–X_2_–X_3_”, in which X_0_ is an aromatic residue (Trp/Phe/Tyr), X_1_ and X_2_ can be any amino acids (often acidic or hydrophobic residues), and X_3_ is a large hydrophobic residue (Leu/Val/Ile) [10]. The main target of AnkB-LIR is GABARAP, since it was demonstrated that the peptide EEWVIVSDEEIEEARQKA binds to it with a K_d_ value of 0.27 nM. However, despite its potency, AnkB cannot be considered a selective mAtg8-binding peptide, because it interacts with all members of the Atg8 family, displaying a high affinity [11]. The atomic details of the AnkB-LIR/GABARAP interaction were disclosed through X-ray studies by Li et al., who demonstrated that GABARAP inhibitors successfully block autophagy in cultured cells [11]. The results of this investigation paved the way to the design of GABARAP binders as potential tools for the development of anticancer drugs. While it is known that peptides are endowed with poor PK properties (because of their low resistance to intestinal degradation), they can still be valuable tools for the study of the physio-pathological pathway in which their biological counterparts are involved. Moreover, they constitute valuable templates for the design of novel small molecules or peptidomimetics.

In this paper, with the aim of identifying new peptides endowed with inhibitory activity against GABARAP, we start from the AnkB-LIR/GABARAP X-ray complex and, by applying a computational approach, we identify new peptides with low micromolar affinity for the target. Experimental assays were carried out to measure their K_d_ values by Microscale Thermophoresis (MST) [12] and SPR, and to evaluate their activity on prostate cancer cells. Remarkably, two of them displayed anticancer effects on PC-3 cells. Considering that prostate cancer is the second most diagnosed malignancy in men worldwide, we are confident that this study will open new avenues to identify the chemical entities endowed with significant therapeutic effects on this widespread disease.

## 2. Results and Discussion

Initially, the GABARAP/AnkB-LIR computational model was generated, starting from the three-dimensional data of the complex, available in the PDB. The simulation, refined by molecular modeling techniques (see Section 3 for details), was then used to design new peptides endowed with high affinity for GABARAP. The following procedure was adopted:

1. Identification of the minimal AnkB-LIR sequence (core) responsible for the interaction with GABARAP;
2. Mutation of the core sequence aimed at improving the theoretical affinity of the resulting peptides;
3. Rigidification of the most promising peptides by adding disulfide bonds;
4. Optimization of the peptide sequence by the addition of residues potentially occupying additional GABARAP binding pockets;
5. Estimation of the binding affinity of the peptides by biophysical experiments;
6. Evaluation of the killing effect on prostate cancer cells, exerted by the most promising candidates.

### 2.1. Setup of the GABARAP Computational Model and the Identification of the AnkB-LIR Core Sequence

**Figure 1:**
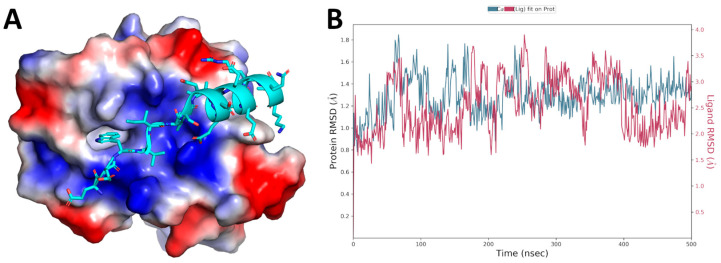
(**A**) 3D representation of the GABARAP/AnkB-LIR complex, as derived from the X-ray structure (PDB accession code 5YIR). The protein surface is colored depending on the atomic par-tial charges of the protein residues: blue for positive and red for negative charges. The AnkB-LIR peptide is represented as cyan sticks. (**B**) Plot of the protein and ligand (AnkB-LIR) Cα atoms RMSD over the simulation time.

The GABARAP/AnkB-LIR complex model (Figure 1A) was retrieved from the Protein Data Bank (PDB) (accession code 5YIR) [11] and then refined by energy minimization and molecular dynamics (MDs) simulations, following the procedure reported in Section 3. The AnkB-LIR peptide rapidly reached the geometrical stability over the 500 ns long MD simulation, as demonstrated by the protein Cα RMSD plot (Figure 1B).

As expected, and verified by inspecting the MD trajectory frames, the LIR domain (sequence WVIV of AnkB-LIR) created numerous contacts with GABARAP (Supplementary Materials Figure S1). Interestingly, the SDEE residues were also involved in productive contacts, including the electrostatic interactions between the side chains of the peptide glutamates and the positively charged area of GABARAP close to K_46_ and R_47_. Conversely, the remaining C-terminal residues were mainly involved in internal contacts, stabilizing the α helix shaped by the DEEIEEARQKA sequence.

Then, to exactly define the minimal portion of AnkB-LIR with the highest affinity for GABARAP, the peptides AnkB-LIR and AnkB-core (sequence WVIVSDEE) were subjected to MD simulations and MM-GBSA calculations to estimate their binding free energy. Desmond and Prime tools of Maestro were employed for these computations, which predicted ΔG* values of −135.1 and −107.9 kcal/mol for AnkB-LIR and AnkB-core, respectively (Table 1). This result indicates that the 8 amino acids belonging to the AnkB-core contribute 75% of the overall interaction energy of the full AnkB-LIR peptide (composed of 20 residues). Then, to further define the contact area, MD simulations and MM-GBSA calculations were performed on the GABARAP/WVIV complex model, in which only the LIR motif (AnkB-wviv peptide) was simulated. As reported in Table 1, residues WVIV contribute 65% of the overall binding free energy. This outcome confirms that the LIR motif, shared by all proteins involved in the autophagy machinery, displays the highest complementarity with the GABARAP-binding site and is responsible for the most significant protein–protein contacts. Subsequently, the same computational protocol (MD simulations and MM-GBSA calculations) was applied to study the interaction of peptide K1. Considering that its experimentally determined K_d_ lies in the nanomolar range (390 nM), this peptide could be considered as a reference inhibitor of GABARAP, together with AnkB-LIR. By our computations, the predicted ΔG* for peptide K1 on GABARAP was −118.9 kcal/mol (Table 1), a value slightly higher than that of AnkB-LIR (−135.1 kcal/mol). This result is in line with the K_d_ values reported for the two peptides (0.27 and 390 nM, respectively).

### 2.2. Computational Design of AnkB-Core Analogs

Considering that the WVIVSDEE (AnkB-core) sequence accounts for 75% of the GABARAP/AnkB-LIR contacts, we proceeded to design small peptides endowed with high affinity for GABARAP using AnkB-core as a template. In this attempt, only the residues of the LIR domain (WVIV, positions 2–5 of AnkB-LIR) of AnkB-core (WVIVSDEE) were mutated, because of their direct involvement in the interaction with GABARAP. In this challenging effort, we tried to optimize the peptide sequence, also shared by other GABARAP binders, to improve the ligand/protein complementarity and selectivity. To this end, the “affinity maturation protocol”, implemented into the Prime module of the Maestro software, was utilized to mutate the VIVS residues into all possible natural amino acid combinations. To avoid the combinatorial explosion, the Monte Carlo optimization option was selected. By this option, 2000 peptides were randomly generated by Monte Carlo algorithm and the peptides with a maximum of three simultaneous mutations were accepted to create the output file containing 100 solutions. Then, the Prime module was also employed to establish whether the mutations led to a more favorable interaction with the biological counterpart, by calculating the mutant peptides binding free-energy (ΔAffinity) values [13].

At the end of these calculations, we visually inspected the results for the first 100 peptides with the highest predicted affinity. Among the predicted peptides, we noted that 7 of them displayed ΔAffinity values lower than 2 kcal/mol with respect to the initial template (AnkB-core). In these peptides, position 2 was substituted by Arg, Glu, or Ile; positions 3 and 4 contained only Ile; while position 5 included only alkaline residues, such as His and Arg. Among them, only one candidate, WEIIHDEE, named Pep-sol4, was further investigated by MD simulations and MM-GBSA calculations, because it presented an interesting Glu in position 2. Through this acidic amino acid, the peptide could potentially interact with the positive area shaped by GABARAP-K_46_ and GABARAP-R_67_ (two conserved residues among Atg8 proteins). Moreover, GABARAP-K_46_ is considered to be a universal gate-keeper, regulating the entrance of ligands interacting through the LIR motif [14]. The structural alignment of the GABARAP/Pep-sol4 complex to the GABARAPL2/UBA5 NMR structure (PDB accession code 6H8C) [15] confirmed that the glutamate in position 2 of Pep-sol4 could reproduce the interaction displayed by E_15_ (GAMEIIHEDNEWGI**E**LVSE) of the “ubiquitin-like modifier activating enzyme 5” (UBA5) with GABARAP-K_46_.

By applying the computational protocol previously adopted for the reference inhibitors, the binding free-energy value of Pep-sol4 was calculated to be slightly lower than that of AnkB-core (−103.3 vs. −101.4 kcal/mol), suggesting that the new peptide could bind GABARAP with a similar binding affinity (Table 2).

**Figure 2:**
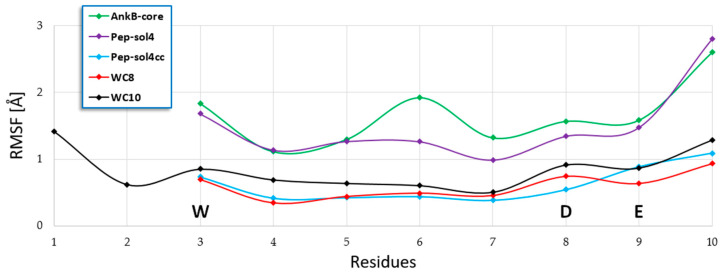
RMSF plots of AnkB-core analogs. Backbone atoms were considered in these calculations. The residues shared by all peptides are highlighted by capital letters.

Moreover, the visual inspection of the GABARAP/Pep-sol4 MD trajectory and the root mean square fluctuation (RMSF) plot of the ligand atoms over the simulation time suggested that the C-terminal portion of the peptide was not firmly bound to the GABARAP surface, thus preventing a stable and productive interaction with the protein (Figure 2). Consequently, considering that the side chains of I_3_ and D_8_ were spatially close in the binding mode adopted by Pep-sol4, we designed a cyclic peptide in which I_3_ and D_8_ were mutated into two Cys residues bound by a disulfide bond. This modification aimed at reducing the conformational flexibility of the ligand, generating a more stable binding mode on the GABARAP surface. The resulting peptide (named Pep-sol4cc, WE**C**IHDE**C**) was again analyzed in the complex with GABARAP by MD simulations and MM-GBSA calculations. At the end of these computational procedures, the estimated ΔG* of Pep-sol4cc was −103.7 kcal/mol, a value comparable to that of Pep-sol4 (−103.3 kcal/mol). This information led us to conclude that the structural rigidification did not affect the affinity of the peptide; however, as demonstrated by the ligand RMSF plot (Figure 2), an improvement of the conformational stability was successfully achieved.

### 2.3. Computational Design of the WC8 and WC10 Peptides

**Figure 3:**
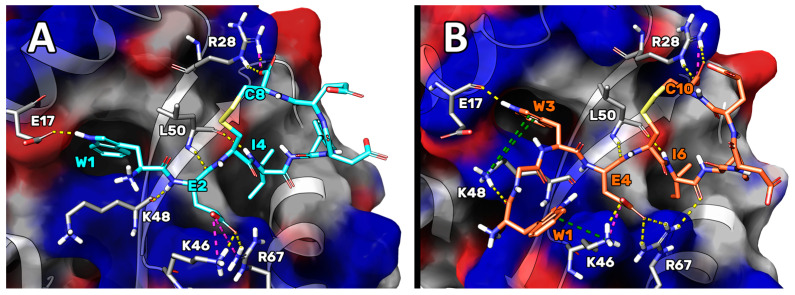
Depiction of the representative structure of the most populated cluster of conformations assumed by WC8 (**A**) and WC10 (**B**) in the complex with GABARAP. The GABARAP solvent-accessible surface is shown accordingly by residue charges: blue for positive and red for negative residues, respectively. The interactions between complexes are represented in colored dashes: yellow for H-bonds, purple for salt bridges, and green for cation-π.

Then, with the aim of improving the theoretical binding affinity of Pep-sol4cc, H_5_ was mutated into a Phe, supposing that it could better fill the hydrophobic area sized by P_52_, L_55_, and Q_59_. The resulting peptide (WC8, sequence WE**C**IFDE**C**) was analyzed by MD simulations and MM-GBSA calculations, which led to a ΔG* value of 12 kcal/mol, lower than that of the originator (Table 2). The cluster analysis performed on the MD trajectory frames displayed that, in the structure representative of the most populated cluster of GABARAP/WC8 conformations (accounting for 79% of conformational ensembles explored), the ligand was stably bound on the GABARAP surface (see Figure 2 for the RMSF plot), forming numerous interactions (Table 3 and Figure 3A).

In detail, several H-bonds were established, and salt bridges formed between WC8-E_2_ and the side chains of GABARAP-K_46_ and -R_67_, and between the C_ter_ of WC8-C_8_ and the side chain of GABARAP-R_28_.

Regarding the hydrophobic contacts, the indole ring of WC8-W_1_ was positioned in a pocket formed by residues I_23_, I_32_, P_30_, K_48_, and F_104_, while the side chain of WC8-I_4_ pointed toward another pocket delimited by Y_49_, V_51_, F_60_, L_63_, and I_64_, establishing van der Waals (vdW) interactions. Finally, similar hydrophobic contacts were also observed between WC8-F_5_ and the GABARAP area shaped by P_52_, L_55_, and L_63_. The complete 2D representation of the interaction network between WC8 and GABARAP is shown in the Supplementary Materials Figure S2A.

WC8 exhibited an estimated ΔG* value close to that of K1; hence, with the aim of designing a more potent peptide, we included two additional N-terminal residues. This hypothesis was supported by the fact that the AnkB-LIR peptide (**EE***WVIVSDEE*IEEARQKA), used as a template, contains two glutamate residues before the AnkB-core (WVIVSDEE). For this reason, we speculated that the homologation of WC8 on the N_ter_ could lead to a more potent peptide, considering that the new atoms could create an additional bond network. Our objective was to reach the region sized by I_32_, Y_5_, and K_47_, close to the W site, on the GABARAP surface (the yellow area in Supplementary Materials Figure S3A). Therefore, to find the optimal *N*-terminal sequence, two glycines were initially added to WC8 (GGWE**C**IFDE**C**); then, the application of the “affinity maturation protocol” on the first Gly residue led to the identification of Tyr (YC10, Table 2) and Trp (WC10, Table 2) as the most suitable substitutions. In this attempt, the glycine in position 2 was not mutated to allow a certain conformational mobility on the *N*-terminal tail of the new peptide. Interestingly, the *N*-terminal residues (WG) and the Glu in position 4 (E_4_) of WC10 (**WG**W**E**CIFDEC) reproduced the interactions displayed by UBA5 (GAMEIIHEDNE**WG**I**E**LVSE) in the complex with GABARAP and GABARAPL2 [15] (Supplementary Materials Figure S3B).

MD simulations and MM-GBSA calculations on the GABARAP/YC10 and GABARAP/WC10 complexes revealed that the latter possessed the highest affinity, with a predicted ΔG* value almost 7 kcal/mol lower than that of WC8 (Table 2). Cluster analysis was then performed on the GABARAP/WC10 MD trajectory frames; the representative structure of the most populated cluster of conformations (which accounts for the 88% of total conformational ensemble explored) is represented in Figure 3B. Notably, the visual inspection of the GABARAP/WC10 most representative structure highlighted that the side chain of the newly added residue (W_1_) formed a cation–π interaction with GABARAP-K_46_, while the carbonyl group of the same residue established an H-bond with the side chain of GABARAP-K_48_ (Figure 3B). Surprisingly, the side chain of W_1_ did not occupy the expected region of GABARAP, but the new additional cation–π interaction greatly contributed to the calculated binding affinity of the peptide. In addition, WC10 (1) shares all the interaction networks established by the GABARAP/WC8 complex, (2) is able to orientate GABARAP-K_48_ in order to establish additional cation–π interactions with WC10-W_3_, and (3) is able to form an additional H-bond interaction between the I_6_(C=O) and GABARAP-R_67_(=NH_2_^+^) (Figure 3). The 2D representation of the interaction network between WC10 and GABARAP is showed in the Supplementary Materials Figure S2B.

To conclude, we designed two new cyclic peptides (WC8 and WC10) endowed with a reduced conformational mobility and calculated binding free-energy values in a lower range than those estimated for the reference peptides, AnkB-core and K1. In light of these data, WC8 and WC10 could exhibit higher experimental affinities compared to the reference peptides. Nevertheless, it must be considered that our computations did not account for the entropic contributions to the binding free energy; hence, they should be regarded as a starting point for further experimental studies.

### 2.4. Experimental Validation and Biophysical Experiments

Based on the results of the computational study, the K1, AnkB-core, WC8, and WC10 peptides were purchased by Proteogenix for the experimental investigations. In detail, MST and SPR assays were conducted on the peptides displaying a sufficient stability in water and PBS buffer. Then, the anticancer potential of the most promising candidates was investigated. Initially, we verified that the peptides were water soluble and stable in the buffer in which the recombinant GABARAP protein was solved. Unfortunately, AnkB-core was not soluble in water; thus, it was impossible to use it as a reference peptide. Conversely, K1, WC8, and WC10 displayed an excellent stability in water and PBS, so they were tested by biophysical methods.

**Figure 4:**
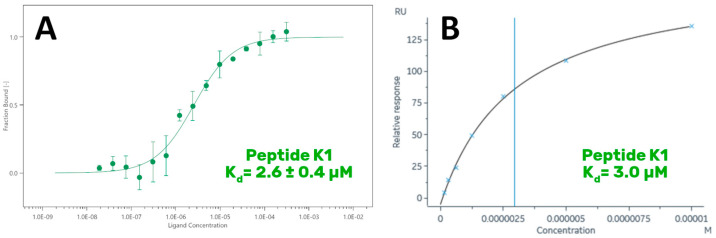
Binding of K1 peptide on GABARAP. MST curve (**A**) and steady-state analysis obtained by fitting the proper form of the Scatchard equation for the plot of the bound RU at equilibrium vs. the ligand concentration in solution during SPR experiments (**B**).

In detail, MST and SPR experiments were carried out with the aim of measuring their K_d_ values on GABARAP. As a preliminary step, the K_d_ of the reference peptide K1 was determined in order to (1) check the experimental procedure and verify the result against data reported in the literature by Weiergräber et al. (K_d_ = 390 nM) [9], and (2) obtain a reference value to compare the K_d_ measured for the new peptides. MST experiments were conducted on a Monolith NT.115 instrument (Figure 4A), while SPR analyses were performed using a BIAcore 8K system, applying the protocol reported by Weiergräber et al. (see Section 3 for details) [9] (Figure 4B).

**Figure 5:**
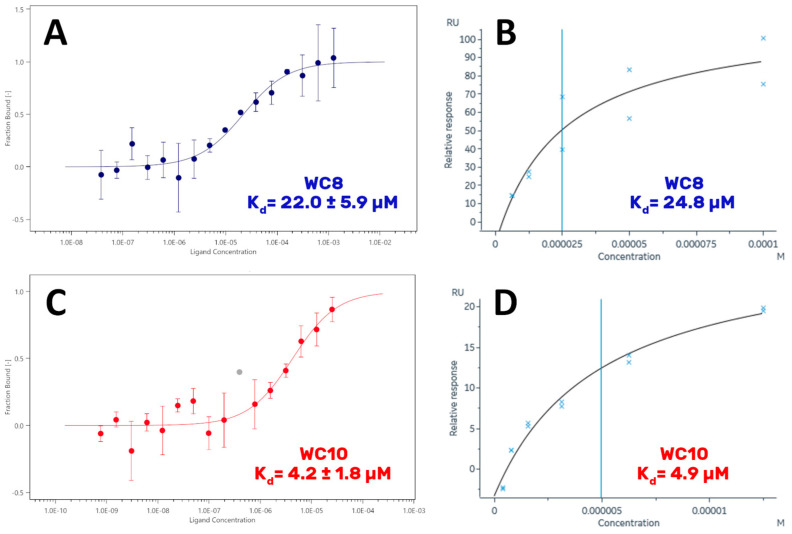
Binding of WC8 and WC10 to GABARAP. MST and SPR curves acquired by recombinant GABARAP incubated with different concentrations of WC8 (**A**,**B**) and WC10 (**C**,**D**) peptides. In the MST plot referred to WC10 (**C**), the point corresponding to a concentration of 391 nM (evidenced in gray) appears to be a clear outlier, also considering the other experiments; hence, it was discarded and not included in the calculation of the K_d_ value.

Surprisingly, the K_d_ measured for K1 was close to 3 µM, a value 7 times higher than the one reported in the literature. However, all the techniques employed in this study agreed on this value. The data obtained for WC8 revealed a K_d_ of 22 µM (Figure 5A,B), consistent among the different biophysical approaches. Remarkably, WC10 displayed a K_d_ in the same range of the reference peptide K1, with a value close to 4 µM, obtained by both MST and SPR (Figure 5C,D).

Because the K_d_ value of peptide K1 proved to be higher than the one reported in the literature, we decided to validate our data by repeating the MST-binding affinity experiments using another Monolith instrument (Monolith NT.115Pico), located in a different laboratory. The new results confirmed our previous findings, with all the peptides displaying K_d_ values consistent with those determined earlier (Supplementary Materials Figure S4).

According to the theoretical predictions, WC10 should be more active than K1 (ΔG* = −122.0 vs. −118.9 kcal/mol, respectively), and WC8 less active than the others (ΔG* = −115.7 kcal/mol), as shown in Table 2. Considering the confidence range of the experimental K_d_ and the omission of the entropic contribution to the estimated binding free-energy values, the computations predicted the affinity trend of the selected peptides well.

### 2.5. Biological Experiments

**Figure 6:**
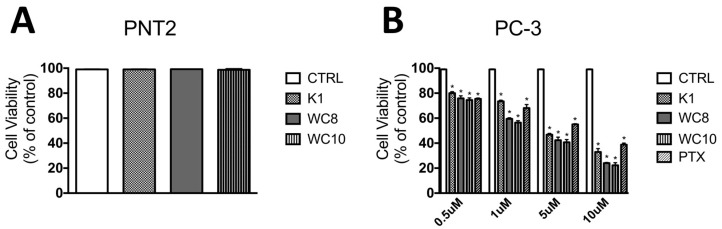
Effect of K1, WC8, and WC10 on cell viability. Cell viability was determined by MTS assay on PNT2 (**A**) and PC-3 cell lines (**B**) 96 h post-treatment. The absorbance was measured with a 96-well-plate spectrophotometer (Varioskan Flash Multimode Reader) at 490 nm.

Finally, K1, WC8, and WC10 were tested in vitro on PC-3 prostate cancer cells, to evaluate their potential antitumor effects (Figure 6). Prostate cancer is the second most commonly diagnosed malignancy in men worldwide. Considering that the probability of developing the disease during a man’s lifetime is 15% and that prostate tumor cells can also spread to the lungs and bones via angiogenesis [16], we decided to evaluate the biological activity of the peptides in vitro on a prostate cancer model. PC-3 cells were chosen for the screening due to their highly metastatic nature, effectively mimicking an aggressive form of the disease. Notably, it has recently been demonstrated that prostate cancer models show a significant upregulation of autophagy [6,8,17].

Therefore, the biological activity of different concentrations of K1, WC8, and WC10 (from 0.5 to 10 µM) was evaluated with an MTS cell viability assay on PC-3 cells and non-cancerous PNT2 prostate cells (Figure 6). The results reported in Figure 6A show that, 96 h post-treatment, none of the tested samples displayed a significant cytotoxicity (cell availability > 90%), confirming the excellent biocompatibility and potential pharmacological selectivity for tumor cells. Indeed, as shown in Figure 6B, a reduction in cell viability (expressed as percentage % of viable cells) was observed in PC-3 cells treated with K1, WC8, and WC10 compared to the untreated control. Interestingly, the treatments of PC-3 cells with WC8 and WC10 (from 1 to 10 µM) display high efficacy, when compared to Paclitaxel (Figure 6B). The in vitro data demonstrate that the compounds exhibited a considerable anticancer activity, especially at the highest tested concentration (cell viability 27.16% for K1, 24.06% for WC8, and 22.5% for WC10).

The biological data on PC-3 cells indicate that all peptides possess IC_50_ values close to 5 µM, consistent with the K_d_ estimated by the biophysical experiments. Surprisingly, WC8 displayed a better activity profile than the reference peptide K1. Based on this finding, we may speculate that some other biochemical mechanism or additional activity on different mAtg8 subfamilies could improve the activity of the new peptides [8]. Nevertheless, since the work presented here is a proof-of-concept study, the peptides have been preliminary tested in a non-cancerous and subsequently in a cancer cell line, to exclude possible off-target cytotoxicity and perform an initial pilot study to evaluate the in vitro anti-cancer efficacy. However, we are planning to extend the screening to other cancer cell lines in the upcoming further evaluation of the peptides and their antineoplastic mechanism of action. Furthermore, to shed light on the possible secondary targets, we have planned biological and biophysical experiments on LC3B to evaluate if our peptides could show any affinity to it. Moreover, we should remember that the data on PC-3 cells represent a preliminary assessment that merely suggests the potential application of GABARAP inhibitors as anticancer agents. Further biological assays are needed to unveil the mechanism by which these peptides trigger cell death.

## 3. Materials and Methods

### 3.1. The GABARAP Computational Model, General Protocol for MD Simulations, and ΔG* Estimation

The computational model utilized in this study was built using the 3D coordinates of the GABARAP/AnkB-LIR complex, available in the PDB, accession code 5YIR [11]. The chains A (GABARAP) and G (AnkB-LIR) were chosen for the creation of the starting complex model. The resulting model was then optimized by the “protein preparation wizard” module implemented in the Maestro software (release 2019-4, Schrödinger, LLC, New York, NY, USA, 2017). This tool permitted us to (1) check the protonation state of the residues at pH 7.4, (2) verify the residue completeness, (3) eliminate atomic clashes, and (4) assign the OPLS3e force field [18]. Then, a cubic box of water molecules (almost 7000, represented by the TIP3P model [19]) was built around the protein–ligand complex and energy minimized by the Desmond algorithm implemented in Maestro. A single run of 500 ns MD simulations was performed, again utilizing the Desmond algorithm, and the “simulation interactions diagram” tool was employed to evaluate the stability of the peptide interacting with GABARAP (see Figure 1B for the GABARAP/AnkB-LIR Cα atoms RMSD variation over the simulation time).

This protocol was also applied for the model setup and MD simulations on the GABARAP complexes resulting from the variation of the length or the mutation of the AnkB-LIR peptide (see Supplementary Materials Figure S5, for the Cα atom RMSD plots of all the other simulated systems). The calculations of the binding free energy values were accomplished by the Prime algorithm [20] available on the Maestro platform, by applying the MM-GBSA algorithm. In these calculations, the single-trajectory approach was applied and the entropy contributions to the binding free energy was neglected. For this reason, the estimated binding free-energy values are termed ΔG* by us. The affinity maturation functionality, implemented in the Bioluminate/Prime module of Maestro, estimated the change in the affinity (ΔAffinity) between the mutant peptides and GABARAP. Finally, the peptides achieving the highest gain in ΔAffinity were additionally refined by MD simulations and MM-GBSA calculations, by applying the previously described protocol. The cluster analysis of the MD trajectories was accomplished by the “Desmond trajectory clustering” tool of Maestro, setting the creation of at least 5 clusters. In these calculations, the RMSD of the backbone atoms was used to create the matrix, which was then analyzed through the affinity propagation clustering method [21].

### 3.2. Microscale Thermophoresis (MST)

The binding affinity (K_d_) between the target protein (6-His-tagged recombinant GABARAP, produced by Abcam, Cambridge, UK) and peptide ligands (K1, WC8 and WC10, acquired by ProteoGenix, Schiltigheim, France) was measured by MST [22]. Briefly, histidine-tagged GABARAP was labeled by using a His-tag-specific dye for 30 min at room temperature, using either the conventional Monolith His-Tag Labeling Kit RED-tris-NTA (MO-L008) or the newer Monolith His-Tag Labeling Kit RED-tris-NTA 2nd Generation (MO-L018), both purchased from NanoTemper Technologies (GmbH, München, Germany). MST experiments were performed on both a Monolith NT.115 and a Monolith NT.115Pico instrument (NanoTemper Technologies, München, GmbH), in order to further increase the statistical significance of the attained results.

A fixed concentration of the labeled GABARAP protein was mixed with 16 1:1 serial dilutions of K1, WC8, and WC10. The protein and the peptide were incubated for 60–90 min at room temperature. The MST analysis was performed using standard capillaries, with the following experimental settings: an MST power of 40% (to create a temperature gradient) and a different excitation power in relation to the considered system and instrument (see Supplementary Materials for details, Table S1). K_d_ values were calculated from compound concentration-dependent changes of normalized fluorescence (Fnorm). In all the experiments, both the protein and the peptides were dissolved in PBS-T buffer (phosphate-buffered saline + 0.05% Tween™ 20; NanoTemper Technologies, GmbH) and 2.5% DMSO. The auto-fluorescence of each peptide was evaluated before proceeding to the determination of the K_d_. At least two independent experiments were performed to calculate the K_d_ values. Data were processed using the NanoTemper MO.Affinity Analysis software (version 2.3), and the fitting was performed by using the K_d_ model.

### 3.3. Surface Plasmon Resonance (SPR)

SPR experiments were performed on a BIAcore 8K system (Cytiva, Marlborough, MA, USA). GABARAP was dissolved in 10 mM of sodium acetate (pH 5.5) and amine-coupled to a CM5 sensor chip (GE Healthcare, Chicago, IL, USA). The reference peptide K1 was dissolved in the running buffer (10 mM Hepes, pH 7.4, 150 mM NaCl) at concentrations of 10 μM to 156 nM. Gradient concentrations of K1 were injected at a flow rate of 30 μL/min, contact time 120 s at 25 °C. WC8 and WC10 were both dissolved in the running buffer (Sigma-Aldrich Dulbecco’s PBS, pH 7.4, 0.005% Tween™ and 5% DMSO; Sigma-Aldrich, St. Louis, MO, USA) at concentrations of 100 μM to 6.25 μM and 12.5 μM to 390 nM, respectively. Gradient concentrations of the peptides were then flowed over the chip with a flow rate of 30 μL/min, contact time 120 s at 25 °C. Solvent (DMSO) correction was applied to the results following the procedure suggested by the instrument producer (BIAcore). Two independent experiments for WC8 and WC10 were performed. The binding kinetics were evaluated using the BIAevaluation software package and applying the 1:1 steady-state affinity model.

### 3.4. MTT Cell Viability Assay

PC-3 prostate cancer cell line was purchased from the American Type Culture Collection (ATCC, Manassas, VA, USA), cultured at 37 °C and 5% CO_2_ in Ham’s F-12 K (Kaighn’s) basal medium (Gibco Laboratories, Grand Island, NY, USA), supplemented with 10% FBS (Gibco Laboratories) and 1% of 100 U/mL penicillin/streptomycin (Gibco Laboratories). The PNT2 (European Collection of Authenticated Cell Cultures, ECACC, UK) human prostate cell line was purchased from Sigma-Aldrich. The cells were cultured at 37 °C and 5% CO_2_ in RPMI 1640 (Gibco Laboratories), supplemented with 10% FBS (Gibco Laboratories), 1% of 100 U/mL penicillin/streptomycin (Gibco Laboratories), and 2% L-glutamine (Gibco Laboratories).

### 3.5. Cell Cytotoxicity Studies

PC-3 and PNT2 cell lines were seeded at a density of 1 × 10^4^ cells/well in 96-well plates and maintained under standard growth conditions. After 24 h, cells were treated with K1, WC8, and WC10 at 0.5 μM, 1 μM, 5 μM, and 10 μM to a final volume of 100 μL; paclitaxel (PTX; Selleck Chemicals) was used as the positive control for the experiments on PC-3 cells.

After 96 h, cell viability was assessed by MTS assay according to the manufacturer’s protocol (Cell Titer 96 Aqueous One Solution Cell Proliferation Assay; Promega, Nacka, Sweden) using a 96-well-plate spectrophotometer (Varioskan Flash Multimode Reader; Thermo-Scientific, Waltham, MA, USA) set at λ = 490 nm. The absorbance value of untreated cells was set at 100% (control), and the viability of treated cells was expressed as a percentage of the control. Three independent experiments were performed in triplicate for each condition.

### 3.6. Statistical Analysis

Statistical significance was analyzed by using Student’s *t*-test. All statistical analyses and calculations were performed using GraphPad Prism (v7.0, 2018, GraphPad Software, San Diego, CA, USA).

## 4. Conclusions

In this study, starting from the GABARAP/AnkB-LIR X-ray crystal structure, we created an affordable GABARAP/AnkB-LIR complex computational model. This was utilized to investigate the role played by different regions of the AnkB-LIR sequence. Then, by applying integrated computational techniques (MD simulations, MM-GBSA calculations, and affinity maturation), we designed two peptides (WC8 and WC10) endowed with theoretical affinities in line with the ones predicted for the reference peptides AnkB-core and K1. The experimental measurement of the affinity values led us to prove that WC10 (2 residues shorter and more rigid than K1) displays a biological activity similar to that of K1. MST, SPR, and in vitro assays on PC-3 cells confirmed this observation. In our opinion, this study has the potential to open new avenues of research towards the design of novel anticancer compounds, employing WC10 as a template. Our results confirm again that a suitable interference with the autophagy process of cancer cells can represent an innovative and viable therapeutic strategy. Consequently, we are confident that the discovery of new potent and specific autophagy modulators will become increasingly important in the near future [23].

## Figures and Tables

**Table 1:** Sequence and calculated ΔG* values of AnkB analogs and K1 peptide.

Peptide	Sequence	ΔG*-Prime ^1^	SD ^2^
AnkB-LIR	EEWVIVSDEEIEEARQKA	−135.1	10.3
AnkB-core	WVIVSDEE	−101.4	5.9
AnkB-wviv	WVIV	−88.5	3.9
Peptide K1	DATYTWEHLAWP	−118.9	10.2

^1^ [kcal/mol]; ^2^ standard deviation [kcal/mol].

**Table 2:** Sequence and calculated ΔG* values of AnkB-core analogs.

Peptide	MW ^1^	Sequence	ΔG*-Prime ^2^	SD ^3^
AnkB-core	976.1	WVIVSDEE	−101.4	5.9
Pep-sol4	1070.1	W**E**I**IH**DEE	−103.3	7.9
Pep-sol4cc	1032.1	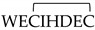	−103.7	4.4
WC8	1042.1	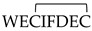	−115.7	4.3
YC10	1262.4	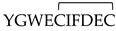	−100.4	5.9
WC10	1285.4	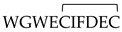	−122.0	5.7

^1^ Molecular weight [g/mol]; ^2^ [kcal/mol]; ^3^ standard deviation [kcal/mol].

**Table 3:** List of the interactions established by the GABARAP/WC8 complex during MD simulations.

WC8	GABARAP (H-Bonds)	GABARAP (Hydrophobic)
W_1_(NH)	E_17_(COO^−^)	I_21_, I_32_, P_30_, K_48_, F_104_
E_2_(COO^−^) *	K_46_(NH_3_^+^) *, R_67_(=NH_2_^+^) *	none
E_2_(C=O)	L_50_(NH)	none
I_4_(NH)	L_50_(C=O)	Y_49_, V_51_, F_60_, L_63_, I_64_
F_5_	none	P_52_, L_55_, L_63_
C_8_(COO^−^_ter_) *	R_28_(=NH_2_^+^) *	none

* Salt bridges.

## Data Availability

Not applicable.

## Supplementary Materials

The following supporting information can be downloaded at: https://www.mdpi.com/article/10.3390/ijms23095070/s1.
