# Supplementary material for: Focused Design of Novel Cyclic Peptides Endowed with GABARAP-Inhibiting Activity
Source: Int J Mol Sci. 2022 May 3;23(9):5070. doi: 10.3390/ijms23095070 (PMC9100651; doi:10.3390/ijms23095070)
Supplement: Supplementary file 1 [file ijms-23-05070-s001.zip › ijms-1693164-supplementary.pdf]

Supporting Materials

Focused Design of Novel Cyclic Peptides Endowed with GABARAP Inhibiting Activity

Enrico Mario Alessandro Fassi [a], Mariangela Garofalo [b], Jacopo Sgrignani [c], Michele dei Cas [a,d], Matteo Mori [a], Gabriella Roda [a], Andrea Cavalli [c], Giovanni Grazioso [*,a]

[a] *Dipartimento di Scienze Farmaceutiche, Università degli Studi di Milano, Via L. Mangiagalli 25, 20133 Milano, Italy.*
[b] *Dipartimento di Scienze del Farmaco, Università di Padova, Via F. Marzolo 5, 35131, Padova, Italy.*
[c] *Institute for Research in Biomedicine (IRB), Via Chiesa 5, 6500 Bellinzona, Switzerland.*
[d] *Dipartimento di Scienze della Salute, Università degli Studi di Milano, Via A. di Rudinì 8, 20146 Milano, Italy.*

* Corresponding author. Phone: +39 02 50319352. E-mail: giovanni.grazioso@unimi.it

Table S1. Summary table of the Microscale Thermophoresis (MST) experiments performed on both Monolith NT.115 and Monolith NT.115Pico instruments. At least two independent experiments were performed to compute the K_d value.

Monolith NT.115							
Protein	Ligand	MST Power	Exc. Power	Temp.	[Ligand] Range	SNR*	K_d (µM)
GABARAP 25 nM	K1	40%	100%	25°C	312.5 µM - 19.1 nM	20.7	2.6 ± 0.4
GABARAP 200 nM	WC8	40%	85%	25°C	1.25 mM - 38.1 nM	12.3	22.0 ± 5.9
GABARAP 25 nM	WC10	40%	80%	25°C	25 µM - 0.8 nM	11.7	4.2 ± 1.8
**Monolith NT.115Pico**							
Protein	Ligand	MST Power	Exc. Power	Temp.	[Ligand] Range	SNR*	K_d (µM)
GABARAP 10 nM	K1	40%	20%	25°C	312.5 µM - 38.1 nM	13.3	3.5 ± 0.9
GABARAP 10 nM	WC8	40%	20%	25°C	625 µM - 38.1 nM	27.6	20.8 ± 2.7
GABARAP 10 nM	WC10	40%	20%	25°C	39.1 µM - 9.5 nM	10.8	3.8 ± 1.5

* Signal to Noise Ratio

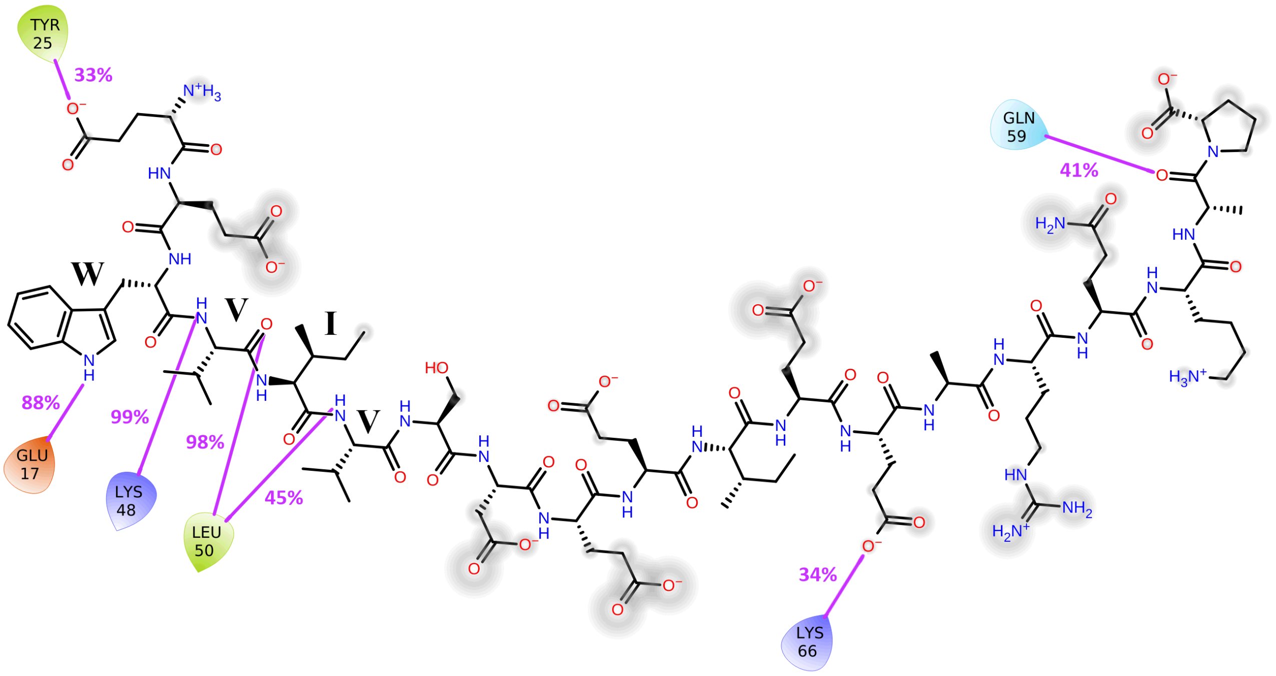

Figure S1. GABARAP H-bond interactions with AnkB-LIR ligand monitored throughout the simulation. The key residues of AnkB-LIR (WVIV) interacting with GABARAP protein are highlighted. Only the interactions that occur more than 30% of the simulation time are shown.

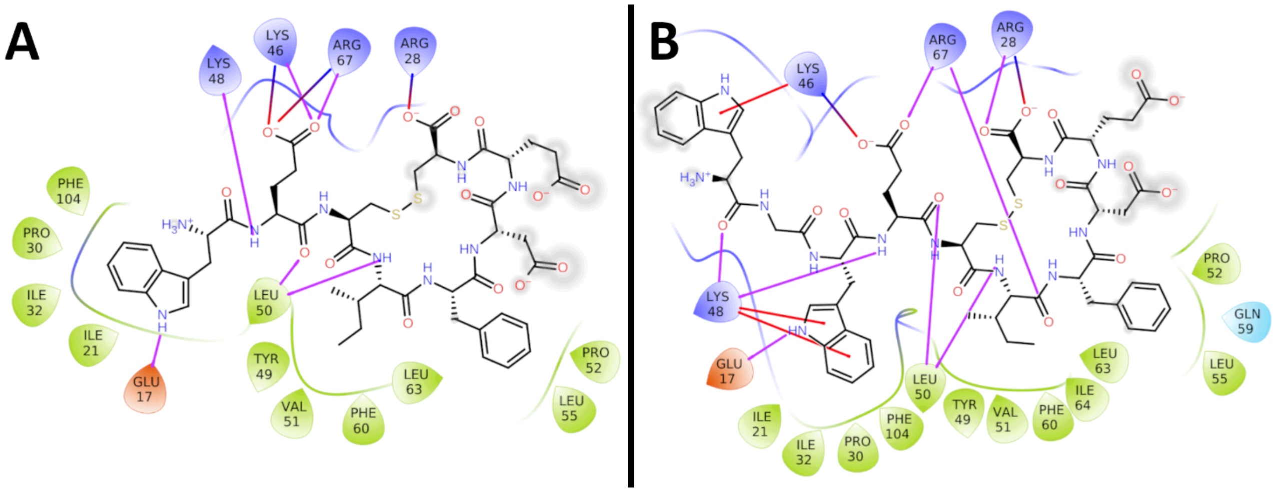

Figure S2. Ligand interaction diagram of WC8 (A) and WC10 (B) peptides with GABARAP protein. H-bond, salt bridges and π-cation interactions are highlighted in purple, blue/red and red lines, respectively. Residues involved in hydrophobic interactions are showed in green.

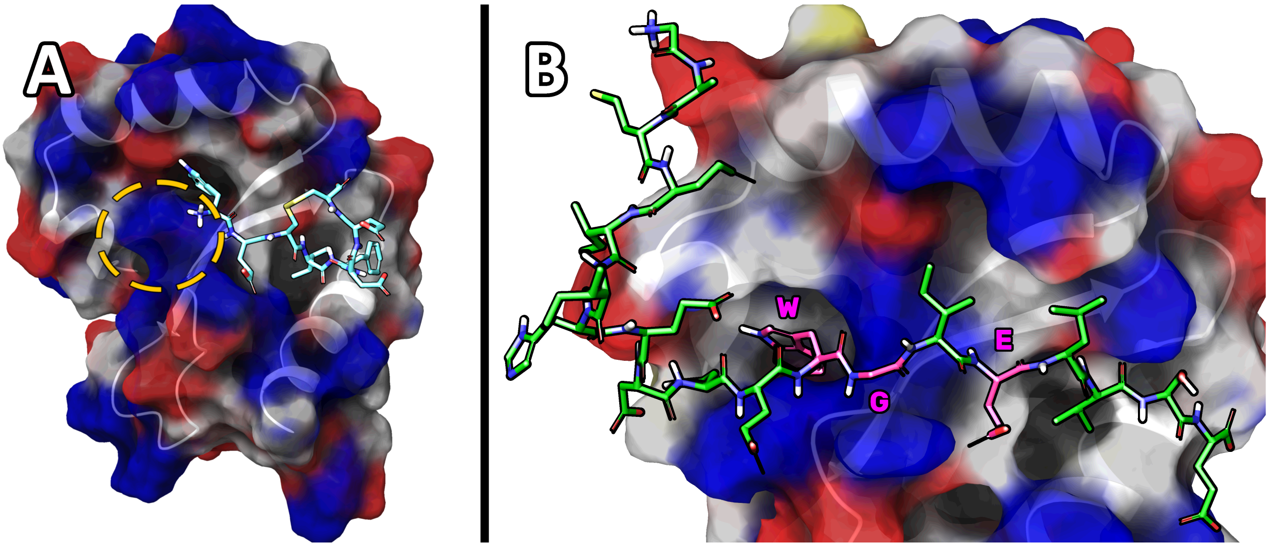

Figure S3. (A) GABARAP/WC8 most representative structure. The yellow circle highlights the protein area in which the two N-terminal residues added on WC8 could be inserted to improve theoretical binding affinity. (B) GABARAPL2/UBA5 NMR structure (PDB accession code 6H8C). In pink sticks are highlighted the residues of UBA5 (GAMEIIHEDNEWGIELVSE) in complex with GABARAP and GABARAPL2 whose interactions are reproduced by peptide WC10 (WGWECIFDEC). The GABARAP solvent-accessible surface is showed accordingly by residue charges: blue for positive and red for negative residues.

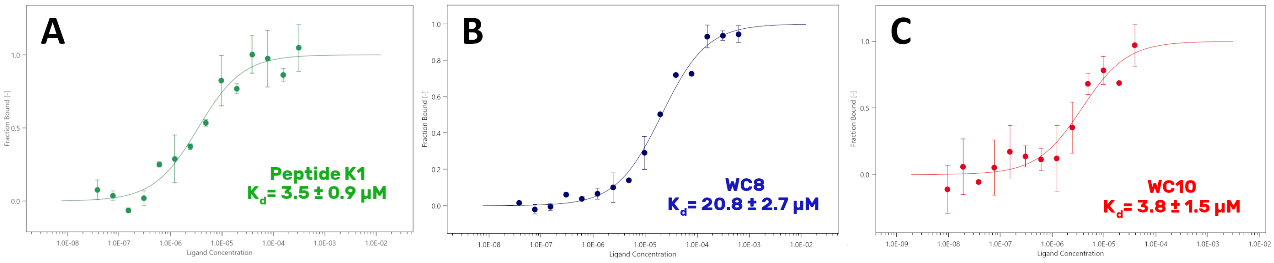

Figure S4. MST curves acquired by GABARAP recombinant protein incubated with different concentrations of K1 (A), WC8 (B) and WC10 (C) peptides using the Monolith NT.115Pico instrument.

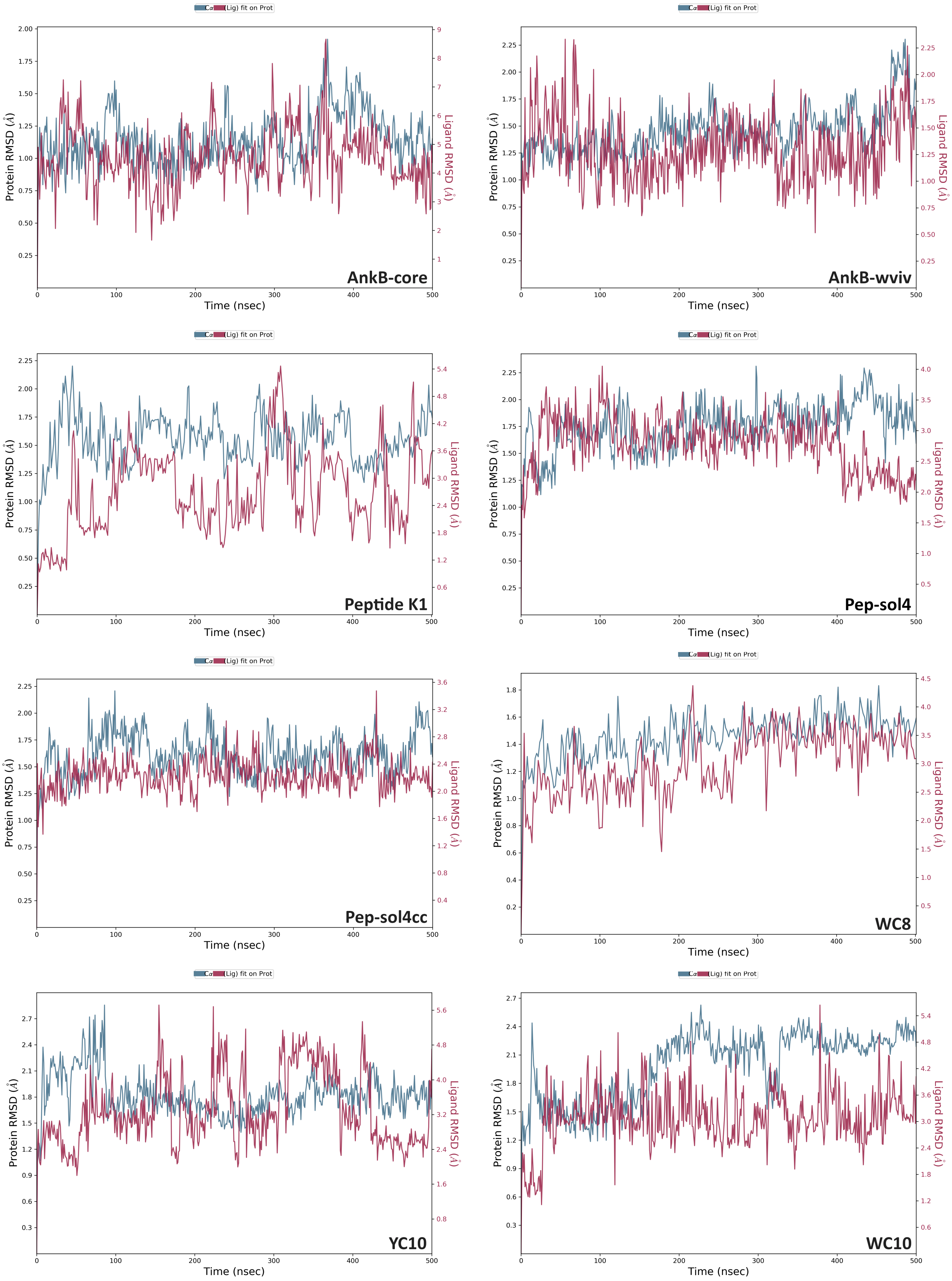

Figure S5. Cα atoms RMSD plots of protein and ligand of the simulated systems over MD simulation time (500 ns).

MASS SPECTROMETRY REPORT
PEPTIDE K1

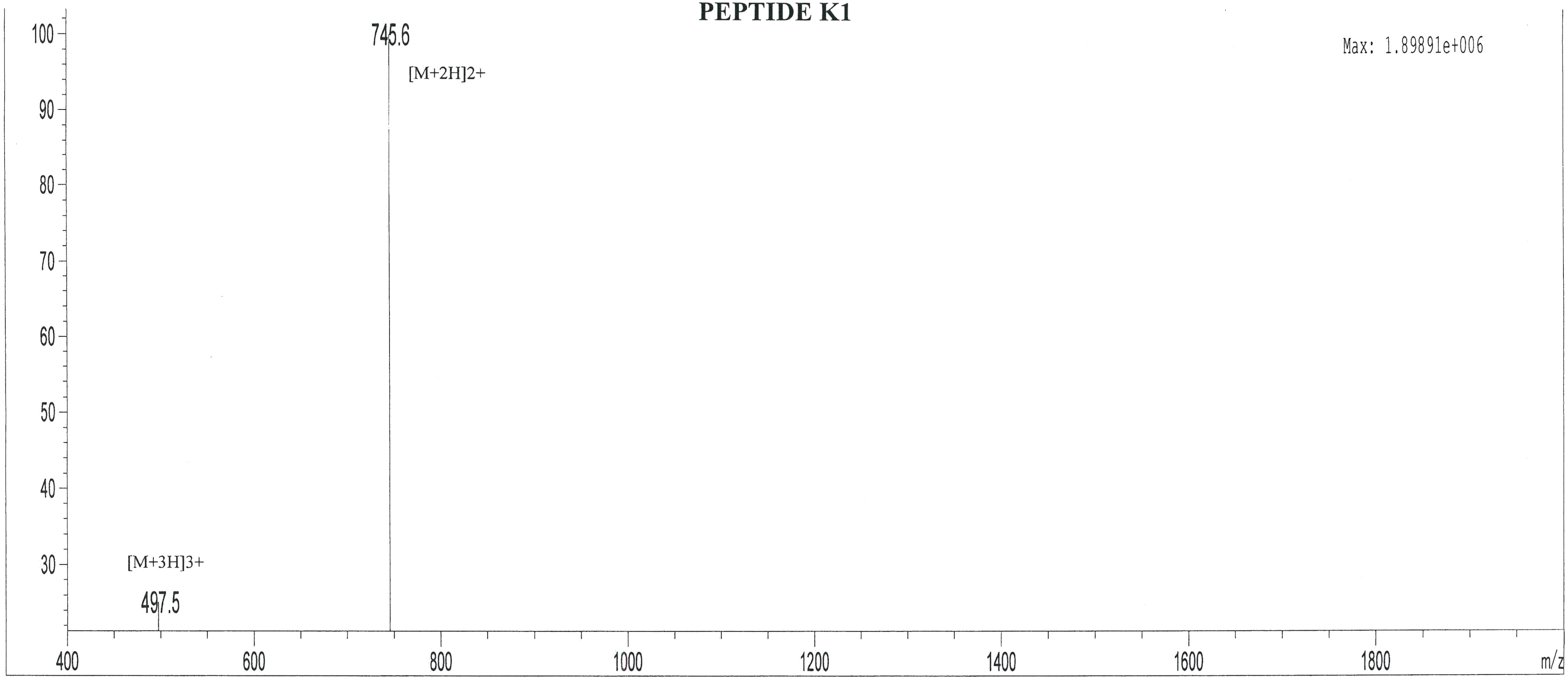

Sample Information		Probe:	ESI	Probe Bias:	+4.5kv
Injection Volume	: 1.00 μ l	Nebulizer Gas Flow:	1.5L/min	Detector:	1.5kv
Sample:	DP-12	CDL:	-20.0v	T. Flow:	0.2ml/min
M.W.:	1489.58	CDL Temp.:	250 °C	B. Conc.:	50%H2O/50%ACN
Lot. No.:	P201104-HS843557	Block Temp.:	200 °C		

MASS SPECTROMETRY REPORT
WC8

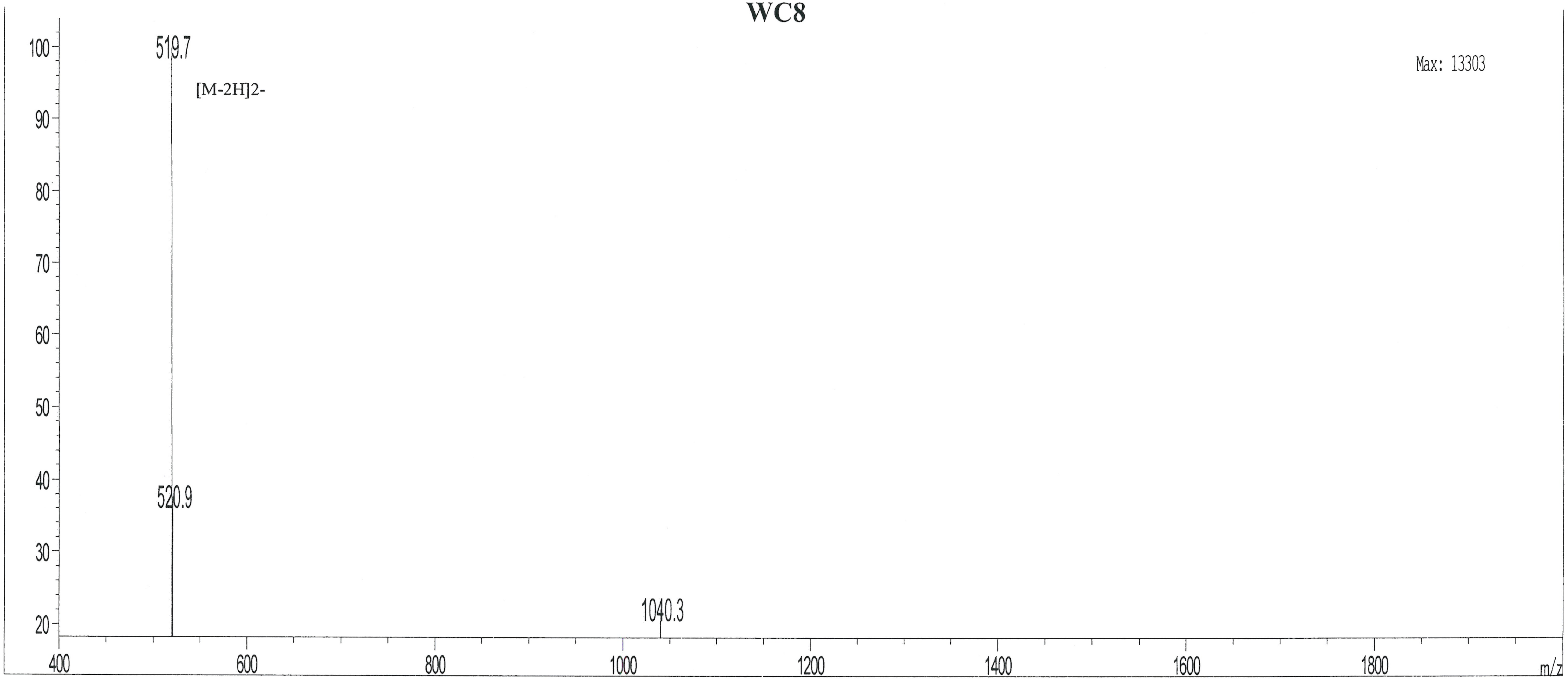

Sample Information		Probe:	ESI	Probe Bias:	+4.5kv
Injection Volume	: 1.00 μ l	Nebulizer Gas Flow:	1.5L/min	Detector:	1.5kv
Sample:	Sol4f-cc WC-8(O)	CDL:	-20.0v	T. Flow:	0.2ml/min
M.W.:	1042.14	CDL Temp.:	250 °C	B. Conc.:	50%H2O/50%ACN
Lot. No.:	P200318-SY788987	Block Temp.:	200 °C		

MASS SPECTROMETRY REPORT
WC10

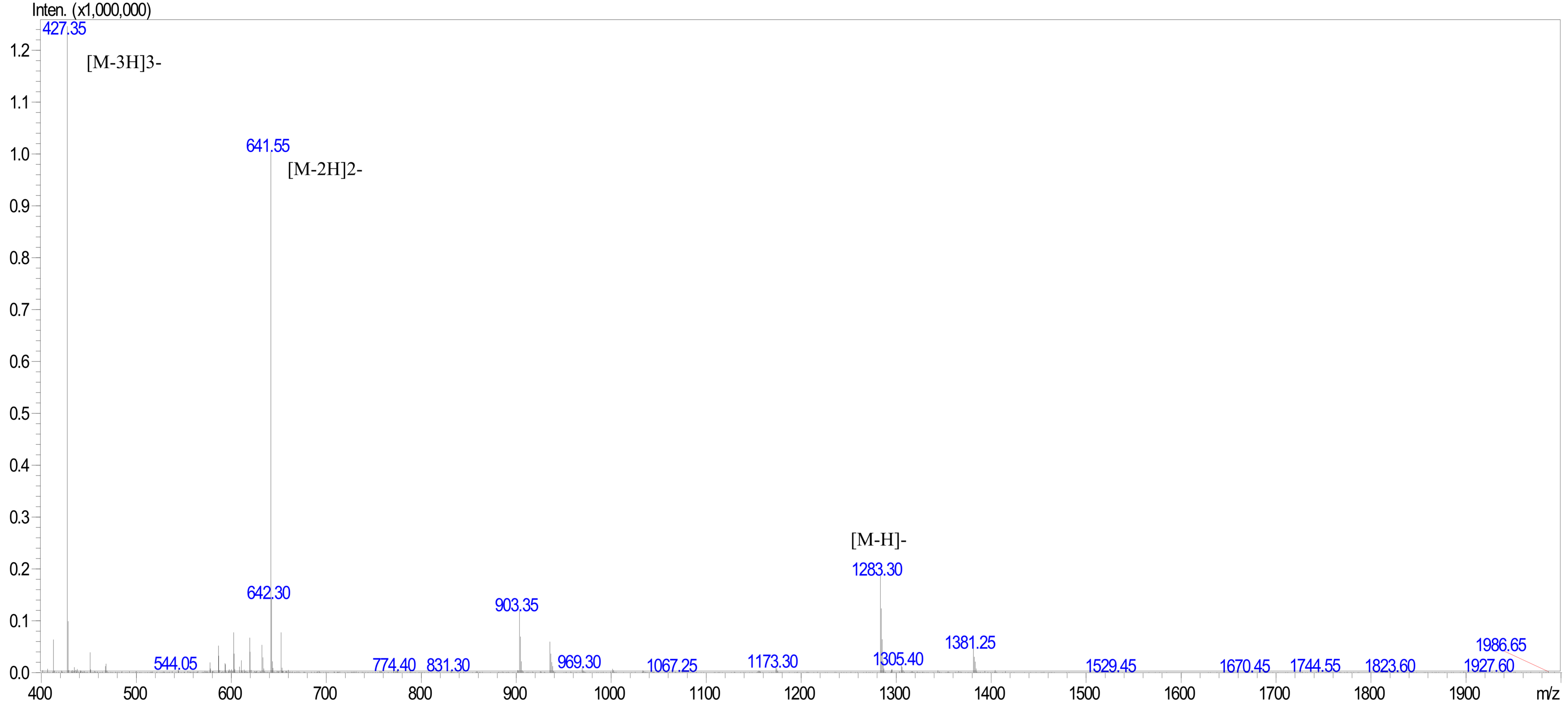

Sample Information		Probe:	ESI	Probe Bias:	＋4.5kv
Injection Volume	: 1.00 µ l	Nebulizer Gas Flow:	1.5L/min	Detector:	1.2kv
Sample:	WC10 WC-10	CDL:	-20.0v	T. Flow:	0.2ml/min
M.W.:	1285.40	CDL Temp.:	250 °C	B. Conc.:	50%H2O/50%ACN
Lot. No.:	P210305-CQ874405	Block Temp.:	400 °C		
